# *Achyranthis radix* Extract Improves Urban Particulate Matter-Induced Dry Eye Disease

**DOI:** 10.3390/ijerph16183229

**Published:** 2019-09-04

**Authors:** Tae Gu Lee, Soo-Wang Hyun, Kyuhyung Jo, Bongkyun Park, Ik Soo Lee, Su Jeong Song, Chan-Sik Kim

**Affiliations:** 1 Clinical Medicine Division, Korea Institute of Oriental Medicine, Daejeon 34054, Korea (T.G.L.) (B.P.); 2 Herbal Medicine Research Division, Korea Institute of Oriental Medicine, Daejeon 34054, Korea (S.-W.H.) (S.J.S.); 3 Non-clinical Research Collaboration Division, Korea Institute of Oriental Medicine, Daejeon 34054, Korea (K.J.) (I.S.L.); 4 Korean Convergence Medicine, University of Science Technology (UST), Daejeon 34054, Korea

**Keywords:** urban particulate matter, dry eye disease, *Achyranthis radix* extract

## Abstract

Dry eye disease (DED) is a multifactorial inflammatory disease that severely impairs patients’ quality of life. Particulate matter comprises a harmful mixture of particles less than 10 μm in size, which on contact with the eye, causes inflammation in the cornea/conjunctival epithelium, threatening eye health and triggering the onset of DED. *Achyranthis radix* is an ingredient of traditional medicine generally used for treating osteoporosis, trauma, and thrombosis in Asian countries. However, the effect of *Achyranthis radix* on eye health has not been elucidated. In this study, we evaluate the protective effect of *Achyranthis radix* hot water extract (ARE) in a rat model of urban particulate matter (UPM)-induced DED. UPM with or without ARE were topically administered on both eyes thrice daily for 10 days. ARE induced tear secretion and improved corneal irregularity. Additionally, ARE treatment protected the corneal epithelial cells from UPM-induced apoptosis. It also restored rMuc4 expression in the cornea and increased goblet cell density in the conjunctiva. These results are suggestive of the potential of ARE as a topical therapeutic agent for treating DED.

## 1. Introduction

Dry eye disease (DED) is an inflammatory ocular disease, which frequently occurs in the elderly and in women [1]. At the Tear Film and Ocular Surface Dry Eye Workshop II, the definition of DED was refined to state as follows: a multifactorial disease of the ocular surface characterized by a loss of homeostasis of the tear film, and accompanied by ocular symptoms, in which tear film instability and hyperosmolarity, ocular surface inflammation and damage, and neurosensory abnormalities play etiological roles [2].

The ocular surface can be exposed to external trauma and harsh environments, such as physical injury, exposure to ultraviolet radiations and particulate matter (PM), which can initiate a vicious inflammatory cycle resulting in DED [3,4]. Physiologically, the ocular surface is protected by a three-layer tear film consisting of lipid, aqueous, and mucin layers. Each layer is maintained by different specialized glands and cells. The superficial lipid layer is secreted by the meibomian glands of the eye lid and blocks tear evaporation and lowers the surface tension to stabilize the tear film [5]. The aqueous layer is secreted by several lacrimal glands that are regulated by parasympathetic and sympathetic nerves [6]. The mucin layer, which acts as a barrier to the entry of pathogens and maintains surface lubrication, is secreted by the corneal epithelial cells, conjunctival epithelial cells, and goblet cells [7,8]. Hence, the homeostatic maintenance of these layers is essential to secure the integrity of the ocular surface.

Due to air pollution, many individuals around the world are at a risk of developing eye, respiratory, and cardiovascular health complications [9]. The presence of PM is one of the critical problems of air pollution; PM is a complex mixture of particles less than 10 μm and is mainly composed of carbonaceous material, metals, polycyclic aromatic hydrocarbon (PAHs), nitrates, sulfates, and biological components [10]. The deleterious effects of PM on components of the ocular system have been well reported in in vitro and in vivo studies [11,12,13,14,15].

*Achyranthis radix*, the root of *Achyranthes japonica Nakai*, a member of the *Amaranthaceae* family, has been used in traditional medicine among Asian countries for treating osteoporosis, trauma, and thrombosis [16]. Experimental research has revealed numerous pharmacological activities of *Achyranthis radix* extract (ARE), such as anti-tumor, immunostimulatory, uterotonic, anti-fertility, cognition-enhancing, anti-bacterial, anti-aging, anti-inflammatory, and anti-osteoporotic activities [17]. However, the biological activities of ARE associated with the ocular homeostatic system have yet to be identified. Therefore, in this study, we examined the protective effect of ARE on the ocular components in a rat model of urban particulate matter (UPM)-induced DED.

## 2. Materials and Methods

### 2.1. UPM Sample

UPM sample (Standard Reference Material 1648a) was purchased from Sigma-Aldrich (St. Louis, MO, USA). The sample was collected for approximately 12 months between 1976–1977 in the St. Louis area of Missouri and was standardized by the National Institute of Standards and Technology (NIST, Gaithersburg, MD, USA). UPM contains PAHs, nitro-substituted PAHs, polychlorinated biphenyls, metals, and inorganic elements.

### 2.2. Preparation of ARE

*Achyranthis radix* was collected in Youngam-gun, Jeollanam-do, Korea, in January 2017, and identified by Ki Hwan Bae, Chungnam National University, Republic of Korea. A voucher specimen was deposited in the herbarium of the Korea Institute of Oriental Medicine (Daejeon, Korea). Five kilograms of dried and ground *Achyranthis radix* was boiled in 40 L of primary distilled water for 3 h at 100 °C. Thereafter, the extract was concentrated by the freeze-drying method into powder form. Concentrated ARE powder was deposited at the Herbarium of Korea Institute of Oriental Medicine (Daejeon, Republic of Korea).

### 2.3. HPLC Conditions

HPLC analysis was performed with an Agilent 1200 HPLC instrument (Agilent Technologies, Santa Clara, CA, USA) equipped with a binary pump, vacuum degasser, autosampler, column compartment, and diode array detector (DAD). The Kinetex C18 column was used (100 × 4.6 mm, 5.0 μm, Phenomenex, Torrance, CA, USA). The mobile phase was a mixture of solvent A (water with 0.1% formic acid) and solvent B (acetonitrile). A linear gradient elution was performed from 5% to 20% B in 20 min and from 20% to 25% B in 10 min, followed by washing and reconditioning the column. Column temperature was maintained at 35 °C. Analysis was performed at a flow rate of 1.0 mL/min and monitored at 250 nm. Standard compound ecdysterone was purchased from Acade Chemical (Kowloon, Hong Kong). Additionally, 25*R*-inokosterone, and 25*S*-inokosterone were purchased from Chengdu Biopurity Phytochemicals (Wenjiang, Chengdu, China)

### 2.4. Animals

Six-week-old female Sprague-Dawley (SD) rats were purchased from Orient Bio (Seongnam, Korea). The animals were supplied with a standard diet and water ad libitum. Rats were housed in specific pathogen-free facilities managed by the Korea Institute of Oriental Medicine, with temperatures of 22–24 °C, relative humidity 50%–60%, and 12 h light/12 h dark cycles. The animal experiments were conducted in accordance with the Institutional Animal Care and Use Committee approved protocol (approval ID. 17-060). 

### 2.5. Animal Experimental Design

The thirty female SD rats were randomly divided into five groups as follows: (Control, *n* = 6), saline; (DED, *n* = 6), 20 mg/mL UPM; (ARE-0.1%, *n* = 6), 20 mg/mL UPM with 0.1% ARE; (ARE-0.5%, *n* = 6), 20 mg/mL UPM with 0.5% ARE; (ARE-1%, *n* = 6), 20 mg/mL UPM with 1% ARE. Twenty microliters solution of UPM (20 mg/mL; dissolved in saline) with or without ARE were topically administered on both the eyes of the experimental animals, thrice daily for 10 days.

### 2.6. Tear Production

Tear production was measured using phenol red threads (Tianjin Jingming New Technological Development, Tianjin, China). The rats were anesthetized by intraperitoneal injection of pentobarbital (40 mg/kg, Entobar; Hanlim pharm. Co., LTD, Seoul, Korea). The phenol red threads were inserted at one-third of the distance from the lateral canthus of the lower eyelid for 1 min. The length of red portion of the thread was measured using a microscope (SZ61; Olympus, Tokyo, Japan), and the values were expressed as tear volume in mm.

### 2.7. Corneal Irregularity Score

Corneal smoothness was assessed using a stereoscopic microscope (SZ61, Olympus, Tokyo, Japan) by measuring the reflection of a ring-shaped slit light on the ocular surface. The corneal irregularity was scored using the following scale: 0, no distortion; 1, distortion in one quadrant; 2, distortion in two quadrants; 3, distortion in three quadrants; 4, distortion in all four quadrants; and 5, severe distortion in which no ring was visible.

### 2.8. Terminal Deoxynucleotidyl Transferase Deoxy Uridine Triphosphate Nick End Labeling (TUNEL)

To investigate apoptosis of the corneal cells because of exposure to UPM, paraffin sections of cornea were stained with DeadEnd apoptosis detection system (Roche, Basel, Switzerland) according to the manufacturer’s protocol. The apoptotic corneal cells were visualized using a fluorescence microscope (Olympus, Tokyo, Japan).

### 2.9. Immunohistochemistry

During necropsy, eyeballs were removed from the rats and fixed in Davidson’s solution (Sigma-Aldrich) at room temperature for 24 h and embedded in paraffin blocks. The blocks were cut into 5 μm sections. The sections were deparaffinized with ethanol and hydrated with water. Antigen was retrieved using incubation into boiling 10 mM sodium citrate (pH 6.0) (Sigma-Aldrich) for 20 min. Then, corneal sections were blocked by CAS-Block™ Histochemical Reagent (Thermo, Waltham, MA USA). After washing the sections with Dulbecco’s phosphate-buffered saline (DPBS), the sections were incubated overnight at 4 °C with anti-MUC4 antibody (Thermo). After incubation, the sections were washed with DPBS and marked with labeled streptavidin biotin (LSAB) kit (DAKO, Santa Clara, CA, USA), and the immunostaining was visualized using diaminobenzidine (DAB) substrate kit (DAKO). Counterstaining of the nuclei was performed using Hematoxylin qs (Vector Laboratories, Inc., Burlingame, CA, USA). To analyze MUC4 level, the intensity of immunostaining per unit area (mm^2^) was examined using ImageJ software (NIH, Bethesda, MD, USA).

### 2.10. Periodic Acid-Schiff (PAS) Staining

To evaluate the number of goblet cells, paraffin sections of conjunctiva were stained with PAS staining system (Merck, Kenilworth, NJ, USA). Thereafter, the sections were imaged using a digital camera DP80 (Olympus, Tokyo, Japan) and the goblet cell numbers in the identical area were counted using ImageJ software (NIH).

### 2.11. Statistical Analysis

Results were analyzed using one-way analysis of variance (ANOVA) with Dunnett’s multiple comparisons test. Data were expressed as mean ± standard error of mean. Values of *p* < 0.05 were considered statistically significant. All statistical analyses were performed using the GraphPad Prism 7.0 software (GraphPad, San Diego, CA, USA).

## 3. Results

### 3.1. HPLC Analysis of ARE

**Figure 1:**
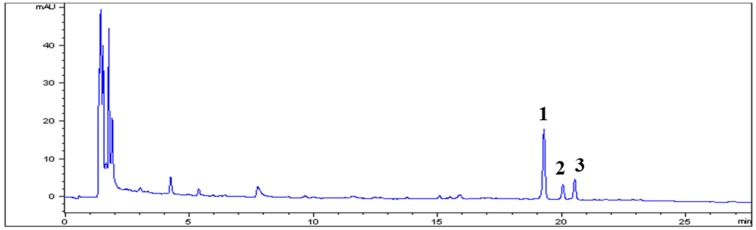
HPLC chromatogram of *Achyranthis radix* extract (ARE). Peak identification: 1, ecdysterone; 2, 25*R*-inokosterone; 3, 25*S*-inokosterone. Chromatographic conditions are described in the text. Detection was at 250 nm.

An HPLC-DAD method was applied for the qualitative analysis of ARE. A typical chromatogram of ARE is shown in Figure 1. Main chromatographic peaks 1, 2, and 3 in ARE appeared at retention times of 19.28, 20.06, and 20.55 min, respectively. Comparison of the retention times and UV spectra (249 nm) of these peaks with those of the corresponding standards confirmed the chemical identities of peaks 1, 2, and 3 as ecdysterone, 25*R*-inokosterone, and 25*S*-inokosterone, respectively.

### 3.2. Administratoin of ARE Restored Tear Secretion

**Figure 2:**
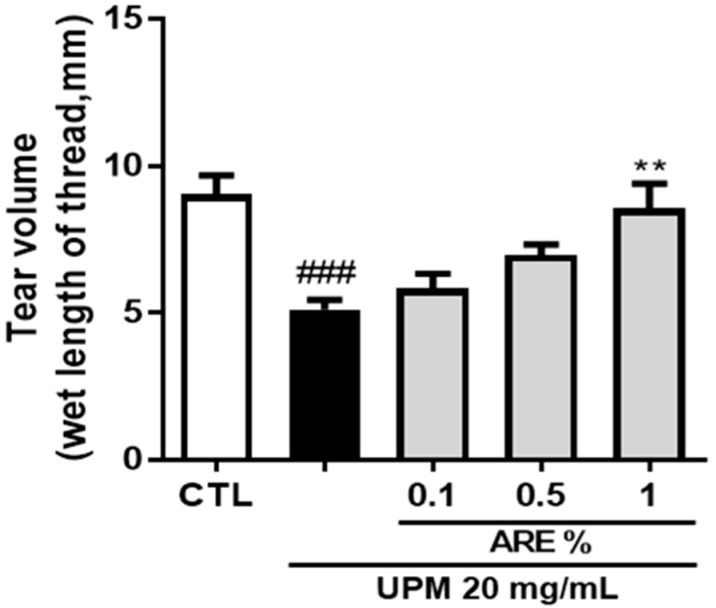
*Achyranthis radix* extract restored tear secretion in rat model of urban particulate matter-induced dry eye disease. The volume of tears secreted was measured using the phenol red thread test. The length of the thread that changed color to red is shown for each group. Each bar represents the mean tear volume ± SEM, *n* = 6. ### *p* < 0.001 vs. control group, ** *p <* 0.01 vs. UPM group. CTL, control; ARE, *Achyranthis* radix extract; UPM, urban particulate matter.

We evaluated the effect of topical administration of ARE on the rat model of UPM-induced DED. As shown in Figure 2, a five-day exposure to UPM caused extreme abrogation of tear production from 9.0 ± 0.6 mm to 5.1 ± 0.3 mm. Subsequently, the tear production was restored on ARE treatment, and significant improvement in tear production was observed in the ARE 1%-treated group (8.5 ± 0.8 mm) than that in the UPM-exposed group. However, no significant improvements in tear production were observed in the ARE-0.1% and ARE-0.5%-treated group.

### 3.3. Administration of ARE Restored Corneal Smoothness

**Figure 3:**
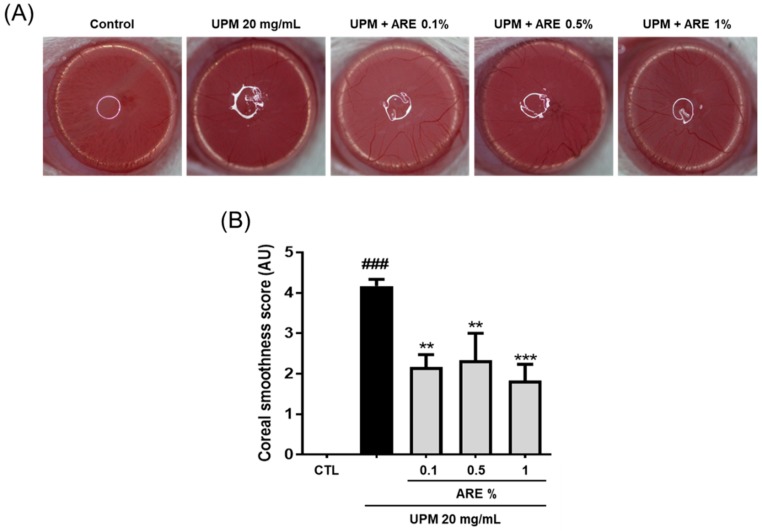
*Achyranthis radix* extract improved corneal smoothness. (**A**) Corneal smoothness was evaluated by the reflection images of the white ring from the light source on the surface of the eye. (**B**) Each bar represents mean corneal irregularity score ± SEM, *n* = 6. ### *p* < 0.001 vs. control group, ** *p* <0. 01, *** *p* < 0.001 vs. urban particulate matter group. ARE, *Achyranthis radix* extract; UPM, urban particulate matter; CTL, control; AU, arbitrary unit.

To evaluate the protective effect of ARE in improvement of the corneal surface smoothness, the UPM-exposed rats were treated with ARE, thrice daily for 10 days. As shown in Figure 3A, UPM seriously distorted the reflection of the ring-shaped white light (Figure 3B). The corneal irregularity score increased in the UPM-exposed (4.2 ± 0.2) group than that in the normal group (0). The corneal irregularity demonstrated significant improvement with all concentrations of ARE treatment (0.1%, 2.2 ± 0.3; 0.5%, 2.3 ± 0.6; 1%, 1.8 ± 0.2), indicating that ARE was beneficial in maintaining the corneal smoothness.

### 3.4. Protective Effect of ARE on the Corneal Epithelial Cells

**Figure 4:**
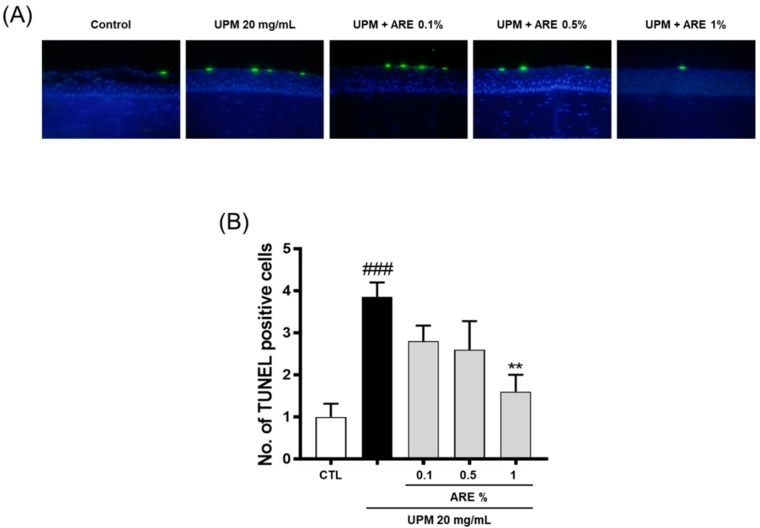
Topical administration of *Achyranthis radix* extract reduced apoptosis of the corneal epithelium. (**A**) Apoptosis of corneal epithelial cell was evaluated using TUNEL assay. (**B**) Each bar represents the mean number of TUNEL-positive cells ± SEM, *n* ≥ 5. ### *p* < 0.001 vs. control group, ** *p* < 0.01 vs. UPM group. ARE, *Achyranthis radix* extract; UPM, urban particulate matter; CTL, control; TUNEL, terminal deoxynucleotidyl transferase deoxy uridine triphosphate nick end labeling; SEM, standard error of mean.

UPM-induced apoptosis of epithelial cells was observed using the TUNEL assay over the corneal surface (Figure 4A). As shown in Figure 4B, the number of TUNEL-positive cells in the UPM-induced DED group was higher than that in the CTL group (CTL, 1.0 ± 0.3; UPM group, 3.9 ± 0.3). UPM-induced apoptosis was significantly reduced in 1%-ARE-treated group (1.6 ± 0.4). However, no significant differences were observed in the UPM-induced apoptosis of corneal epithelial cells in the 0.1- and 0.5%-ARE-treated groups.

### 3.5. ARE Restored rMuc4 Expression on Corneal Surface

**Figure 5:**
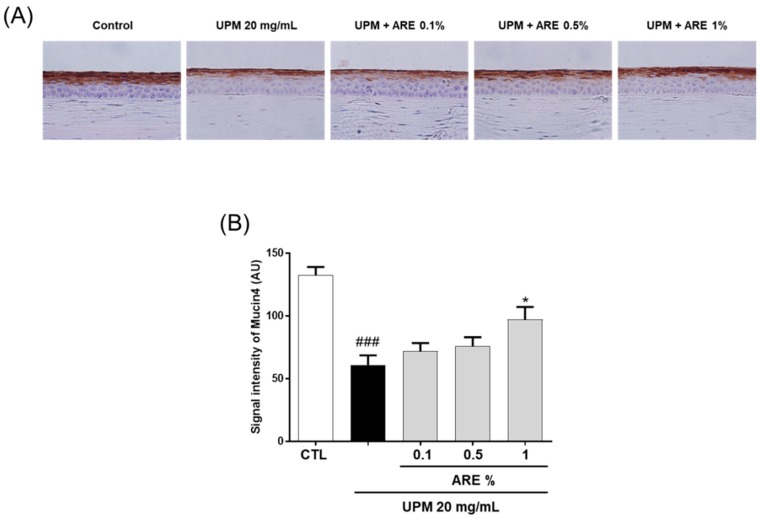
*Achyranthis radix* extract restored rMuc4 expression in the corneal epithelium. (**A**) Immunohistochemical staining of the corneal epithelium was performed by immunostaining the paraffin-embedded corneal sections for rMuc4. The immunostained sections were counterstained with hematoxylin. (**B**) Each bar represents mean rMuc4 fluorescence signal intensity ± SEM, *n* ≥ 5. ### *p* < 0.001 vs. control group, * *p* < 0.05 vs. UPM group. ARE, *Achyranthis radix* extract; UPM, urban particulate matter; CTL, control; AU, arbitrary units; SEM, standard error of mean.

The expression of membrane-bound rMuc4 was analyzed using immunohistochemical staining of the paraffin-embedded corneal sections (Figure 5A). In the UPM group, the expression of rMuc4 was significantly abrogated than that in the CTL group (CTL, 132.8 ± 5.7; UPM group, 60.9 ± 7.0), and this pattern demonstrated significant reversal in the 1% ARE-treated group (97.2 ± 9.1). However, no significant changes were observed in the membrane expression of rMuc4 in the 0.1%- and 0.5%-ARE-treated groups (Figure 5B).

### 3.6. ARE Increased Goblet Cell Density

**Figure 6:**
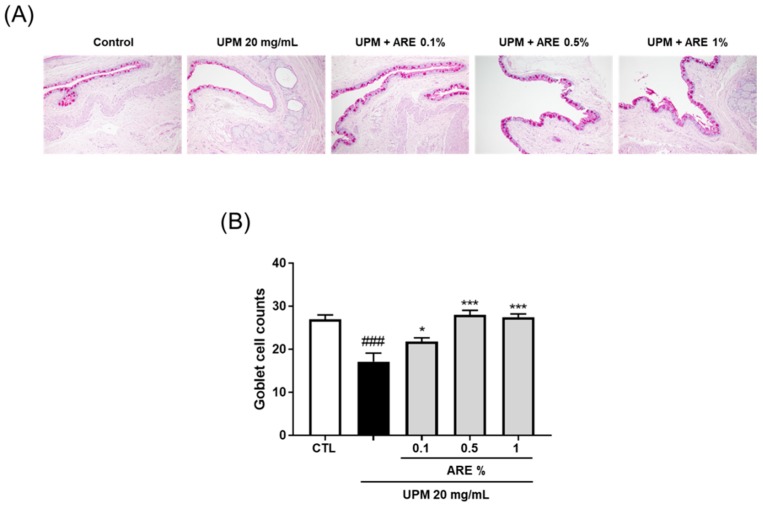
*Achyranthis radix* extract restored goblet cell population in the conjunctival epithelium. (**A**) Representative PAS-stained images of conjunctival sections. The violet colored cells are goblet cells. (**B**) Each bar represents the mean number of goblet cells ± SEM, *n* ≥ 3. ### *p* < 0.001 vs. control group, * *p* < 0.05, *** *p* < 0.001 vs. UPM group. ARE, *Achyranthis radix* extract; UPM, urban particulate matter; PAS, periodic acid-Schiff; CTL, control; SEM, standard error of mean.

Goblet cells are involved in the production and secretion of soluble type rMuc5AC into the tear film. PAS staining was conducted to measure the goblet cell density in the conjunctival epithelium (Figure 6A). PAS-positive goblet cells were found to be extremely decreased with UPM exposure as compared to that in the CTL group (CTL, 27 ± 0.8; UPM group, 17.2 ± 1.8). However, the goblet cell number was found to be significantly restored in all ARE-treated groups (0.1%, 21.8 ± 0.8; 0.5%, 28 ± 0.9; 1%, 27.5 ± 0.7, Figure 6B).

## 4. Discussion

DED is considered as a relatively minor disorder. However, the number of patients with DED is increasing globally, with reports indicating that between 5%–35% of adults worldwide, 17% of women, and 11.1% of men in the United States experience DED [1,18]. DED is a multifactorial disease triggered by various stimuli, such as age, gender, autoimmune disease (Sjögren’s syndrome), dry atmospheric environment, prolonged exposure to or use of digital display, use of contact lens, and chemical exposure (benzalkonium chloride, etc.) [18].

DED is divided into the following two types depending on its pathogenesis: reduced tear production or increased evaporation types [19]. It is well-known that the pathogenesis of DED involves a vicious cycle of inflammation resulting in instability of the tear film, hyperosmolarity, and apoptosis of the corneal/conjunctival epithelial cells on the ocular surface [4]. The initiation of inflammation facilitates the recruitment of more immune cells, which aggravates the symptoms of DED via the production of pro-inflammatory mediators.

PM is considered as one of the risk factors for DED. Some epidemiological studies indicate that air pollution, especially exposure to PM, increases emergency department visits with complaints of conjunctivitis even during non-pollen season [20,21]. Mice subjected to continual instillation of PM-infused eye drops presented DED phenotypes, accompanied by reduced tear secretion, corneal surface damage, increased inflammation of the corneal epithelium and thinner epithelium, reduced goblet cell density, and corneal structural abnormalities [13,15,22]. PM is a complex mixture with varying composition dependent on the location, season, time, and temperature of collection. Thus, it is an unstandardized material whose harmful effects and mechanisms of action are very difficult to evaluate. In this study, we used the Standard Reference Material UPM, NIST 1648a to eliminate uncertainties arising from the collection of PM. Although the mechanism leading to inflammatory stimulus by UPM has not been completely elucidated, heavy metals and PAHs, the major components of UPM, have been reported to bind to toll-like receptors (TLRs) or aryl hydrocarbon receptor (AHR), which subsequently enhances the Th17 polarization of Th cells [10,23]. Moreover, UPM primes macrophages to generate a hyperinflammatory response to lipopolysaccharide [24]. It induces cell death and increases the secretion of inflammatory cytokines or reactive oxygen species in A549 and raw264.7 cells [25,26].

In this study, we identified the protective effect of ARE in an in vivo rat model of UPM-induced DED. The promotion of tear volume is the main therapeutic goal for treating cases of DED. The infiltration of CD4^+^ T cells into the lacrimal glands or the interruption of neuronal stimulation can decrease tear production [27]. Administration of 1% ARE significantly restored the tear volume in the rat model of UPM-induced DED. Moreover, rats treated with lower doses of ARE showed a tendency for an increase in tear volume (Figure 2). The aqueous layer is the largest portion of the tear film and contains many dissolved growth factors, defense proteins, and anti-inflammatory proteins [28]. Thus, the recovered tear volume following ARE treatment seems to have affected the improvement of corneal irregularity (Figure 3).

In a PM-rich atmosphere, eyes are exposed to PM, which dissolves and accumulates in the tear film, and causes cell damage, inflammation, and oxidative stress of the corneal epithelium [11]. In addition, PM induces apoptotic cell death and inflammation via mitochondrial malfunction in cultured corneal and conjunctival cells [29,30,31]. Similarly, UPM induces corneal epithelial cell apoptosis. In this study, the apoptotic death of corneal cells reduced after the administration of eye drops infused with 1% ARE, but no significant changes were observed with the administration of 0.1% and 0.5% ARE (Figure 4).

The apical side of the corneal epithelial cells of the ocular surface express three membrane-spanning forms of mucins (mucin1, mucin4, and mucin16). These molecules confer anti-adhesive, lubricant, water retention, and anti-allergic functions, and moreover, act as a barrier to the entry of pathogens to the ocular surface [32]. Expression of mucins can be altered by inflammatory mediators in the tears of patients with DED [33]. Unlike humans, who do not express extensive MUC4 in the central cornea, rats express rMuc4 in the conjunctiva and cornea [32]. Thus, we evaluated the expression of rMuc4 on the ocular surface despite MUC4 not being the best (MUC1 is best) isoform for clinical detection of patients with DED [34]. On the corneal surface, rMuc4 expression was restored by administration of 10 mg/mL of ARE (Figure 5). Goblet cells in the conjunctival epithelium are the major source of the soluble mucin, Muc5AC. The levels of Muc5AC decreases in patients with Sjögren syndrome or other types of DED because of the abrogation of the function of the goblet cell [35]. In the present study, administration of ARE recovered goblet cell density at the conjunctival epithelium (Figure 6). These results support the fact that ARE demonstrates a beneficial effect on the improvement of clinical markers of DED. 

ARE has numerous molecules, such as triterpenoid saponins, phytoecdysones, polysaccharides, and many other compounds which confer a wide range of medicinal bioactivity [17]. Among these components, saponins and phytoecdysones (ecdysterone and inokosterones) exert anti-inflammatory effects [17]. Especially, ecdysterone has a proven anti-apoptotic effect on sodium arsenite-induced apoptosis of endothelial cells [36]. Moreover, it suppresses IL1β-induced apoptosis and inflammation via the inhibition of NF-κB signaling pathway in chondrocytes [37]. Since DED is an inflammatory disorder, the previously known anti-inflammatory effects of ARE components may be involved in mediating the beneficial effects of ARE identified in this study. However, the bioactive molecule and the mechanism mediating these anti-inflammatory effects are yet to be identified, which warrants further isolation of individual molecules in future studies.

## 5. Conclusions

In the present study, we investigated the protective effect of ARE in a rat model of UPM-induced DED. ARE ameliorates the symptoms of DED by restoring the tear volume, improving corneal surface irregularity, inhibiting corneal epithelial cell death, and increasing rMuc4 expression and goblet cell density. Therefore, we suggest that ARE has therapeutic potential for improving the symptoms of DED.
